# New Insights into CO_2_ Adsorption on Layered Double Hydroxide (LDH)-Based Nanomaterials

**DOI:** 10.1186/s11671-018-2471-z

**Published:** 2018-02-20

**Authors:** Nian Tang, Tingyu He, Jie Liu, Li Li, Han Shi, Wanglai Cen, Zhixiang Ye

**Affiliations:** 1 Electric Power Research Institute of Guangdong Power Grid Company Limited, No. 8 Shuijungang, Dongfengdong Road, Guangzhou, 510080 Guangdong People’s Republic of China; 2 College of Resources and Environment, Chengdu University of Information Technology, No. 24 Block 1, Xuefu Road, Chengdu, 610225 Sichuan People’s Republic of China; 3 Air Environmental Modeling and Pollution Controlling Key Laboratory of Sichuan Higher Education Institutes, No. 24 Block 1, Xuefu Road, Chengdu, 610225 Sichuan People’s Republic of China; 4 Institute of New Energy and Low-Carbon Technology, Sichuan University, Chuanda Road, Shuangliu County, Chengdu, 610207 Sichuan People’s Republic of China

**Keywords:** Modified LDHs, Weakly adsorption, CO_2_ capture, DRIFTS

## Abstract

The interlamellar spacing of layered double hydroxides (LDHs) was enlarged by dodecyl sulfonate ions firstly, and then, (3-aminopropyl)triethoxysilane (APS) was chemically grafted (APS/LDHs). The structural characteristics and thermal stability of these prepared samples were characterized by X-ray diffraction (XRD), transmission electron microscopy (TEM), reflectance Fourier transform infrared spectrometer (FTIR), thermogravimetric analysis (TG), and elemental analysis (EA) respectively. The CO_2_ adsorption performance was investigated adopting TG and diffuse reflectance infrared Fourier transform spectroscopy (DRIFTS). The results presented that the CO_2_ adsorption capacity on APS/LDHs was as high as 90 mg/g and showed no obvious reduction during a five cyclic adsorption-desorption test, indicating its superior performance stability. The DRIFTS results showed that both carbamates and weakly bounded CO_2_ species were generated on APS/LDHs. The weakly adsorbed species was due to the different local chemical environment for CO_2_ capture provided by the surface moieties of LDHs like free silanol and hydrogen bonds.

## Background

The greenhouse effect and global climate change mainly caused by the substantive CO_2_ emission from coal-fired power plants have aroused general public concern [1–3]. Thus, CO_2_ capture should be adopted for its subsequent storage or utilization to reduce its concentration level in the atmosphere. Various solvent- and solid-based sorbents have gained great attention for CO_2_ capture, especially the amine-modified porous material composites for their comparatively low energy consumption for regeneration and easy implementation over a wide range of temperatures and pressures [4–6].

A high and stable CO_2_ adsorption capacity is principal for the large-scale separation process of carbon capture from the flue gas with a large volume and low partial pressure at the temperature range of 50~100 °C [1]. As the amino groups show affinity to CO_2_ molecules, various porous supports with large surface area and pore volumes were adopted to obtain composite adsorbents based on their accommodation to the impregnated amines since Song’s work in 2002 [7]. And the highest CO_2_ adsorption capacity reported in literatures could achieve up to 7.9 mmol/g [8]. However, these kinds of impregnated composites are susceptible to performance degradation during the cyclic adsorption-desorption operation, suggesting a poor stability which is also an important criterion for real applications [9]. Furthermore, strong diffusion limitation is also generated by the agglomerated amines and coated particles for CO_2_ from the surface into the bulky amino groups, which would lower the amine efficiency defined as the amounts of adsorbed CO_2_ molecules for each mole of nitrogen atom.

To improve the thermal stability and amine efficiency of the composite adsorbents, a monolayer or less of CO_2_ affinity sites is formulated via grafting of aminosilanes onto the support materials based on their co-condensation reaction, which has been extensively studied adopting 3-aminopropyltrimethoxysilane (APS), 3-(trimethoxysilyl) propylethylenediamine(diamine) or 3-[2-(2-aminoethylamino)ethylamino] propyltrimethoxy-silane (triamine), etc. [2, 10–12]. These kinds of grafted composites show lower diffusion limitations and superior stability, even though there may be an upper bound of CO_2_ adsorption capacity as it is assumed that two moles of exposed amine groups were demanded to capture one mole of CO_2_ molecules according to the zwitterion mechanism [13, 14]. Nevertheless, the chemical nature of support materials could also affect the CO_2_ adsorption performance. The contributions of surface hydroxyls to CO_2_ capturing either through their direct weak physical force [15] or via its hydrogen bonding with the grafted amines [13, 16] on silica-/titania-based adsorbents have been investigated.

Layered double hydroxides (LDHs) are ordered compounds that assemble by interlamination anions and positively charged layer laminates with the general formula of [M_1 − *x*_^2+^M_*x*_^3+^(OH)_2_]^*x*+^(A^*n*−^)_*x*/*n*_·mH_2_O, where M^2+^ and M^3+^ represent metal cations and A is an anion [17]. LDHs have extended applications in adsorption, catalysis, photochemistry, etc. due to their tunable structure and low cost of raw materials [6, 18–20], which also make it a possible candidate for post-combustion CO_2_ capture. Wang et al. [21] synthesized amine-modified LDHs via an exfoliation and grafting route and reported that these adsorbents would be useful in CO_2_ capture processes with a high temperature of 80 °C, while the CO_2_ adsorption capacity on amine-modified hexagonal mesoporous silica (HMS) decreased from 1.34 mmol/g at 25 °C to 0.45 mmol/g at 75 °C [22]. This suggests the local chemical of LDHs as support materials might affect the CO_2_ adsorption in a way that is different from silica supports. However, few reports have discussed about this as far as we are acknowledged. A systematic study is necessary to investigate the LDH-based sorbents and to further understand their CO_2_ adsorption mechanisms.

With this in mind, (3-aminopropyl)triethoxysilane (APS)-modified LDHs (APS/LDHs) have been prepared in this paper adopting dodecyl sulfate (DS) for pre-intercalation. The structural characteristics of APS/LDHs have been addressed in details elsewhere [21]. Yet some quantities will be reintroduced to illustrate the linkage between CO_2_ adsorption performance and the surface features of LDHs. The CO_2_ adsorption-desorption properties on APS/LDHs were explored mainly using in situ diffuse reflectance infrared Fourier transform spectroscopy measurements (DRIFTS).

## Methods

All the utilized chemicals were purchased from Aladdin reagent Co., Ltd., and were A.R. grade. And these chemicals were used without further treatment.

The as-synthesized LDHs were prepared as the control sample through a co-precipitation method. A mixed solution containing 0.075 mol Mg(NO_3_)_2_·6H_2_O and 0.025 mol Al(NO_3_)_3_·9H_2_O was firstly obtained, which was then added to a Na_2_CO_3_ aqueous solution (0.5 mol/L, 100 mL) under vigorous stirring at 70 °C. The pH value of this mixture was maintained at about 10 using NaOH aqueous solution (4 M) followed by stirring for another 4 h. After that, the resulted precipitate was filtered, washed with distilled water for several times, and dried under vacuum condition at 120 °C overnight. The DS-intercalated LDHs (DS/LDHs) were synthesized 7according to a previous report [21]. Typically, 4.00 g of Mg(NO_3_)_2_·6H_2_O and 1.95 g of Al(NO_3_)_3_·9H_2_O were dissolved in 50 ml of deionized water. The obtained solution was added dropwise into the aqueous solution of sodium dodecyl sulfate (3 g/100 mL distilled water) under continuous stirring at 70 °C. Special attentions should also be paid to the pH adjustment to around 10. The same post-treatment described above was applied to DS/LDHs too.

APS-grafted LDHs (APS/LDHs) were prepared as follows. Two grams of DS/LDHs was dissolved in a conical flask with 500 mL toluene solvent and then subjected to sonication for 5 h. Abundant white gel was gathered half an hour later. Fifteen milliliters of (3-aminopropyl)triethoxysilane was added, and the mixture solution was aged at 60 °C under a nitrogen atmosphere for 5 h. The sediment was extracted by filtration, washed repeatedly, and then dried in a vacuum oven at 120 °C overnight.

The crystal phases of the samples were analyzed by an X-ray diffractometer with CuKα radiation (XRD: model D/max RA; Rigaku Co., Japan, CuKα radiation 0.15418 nm), and data were collected for scattering angles (2*θ*) which ranged from 5° to 70° with a step size of 0.02°. The micromorphology were investigated by transmission electron microscopy (TEM: Tecnai G2 F20; FEI Company, USA). Elemental analysis was performed on Flash EA1112 (Thermo Finnigan, USA). Fourier transform infrared spectrometer (FTIR, IR Affinity-1; SHIMADZU, Japan) was employed to record the IR spectra of the prepared samples. Potassium bromide (KBr) plates mixed with 1/50 of sample were made by applying 20 tons of oil pressure and then scanned from 400 to 4000 cm^−1^ with a resolution of 0.2 cm^−1^. The thermal stability of solid samples was determined using thermogravimetric analysis (TG; NETZSCH STA 409 Luxx, Selb/Bavaria, Germany). The samples were heated from room temperature to 600 °C with a heating rate of 10 K/min in nitrogen atmosphere.

The information about surface species and molecular behavior on the surface of adsorbents were obtained through DRIFTS (Nicolet 6700 FT-IR spectrometer, Thermo Scientific, USA), which was equipped with a temperature control system and coupled with ZnSe windows. In the DRIFTS cell, powder adsorbents were pretreated for 1 h at 200 °C under a nitrogen flow of 30 mL/min. When the temperature was stabilized at 50 °C, a CO_2_ gas flow of 5 mL/min was introduced into the cell for a span of time until complete saturation was achieved. Here, the CO_2_ concentration (~ 14v%) in the gas mixture indicated its general concentration of the industrial flue gas. DRIFTS spectra were collected with 4 cm^−1^ resolution and 64 co-added sans with the consideration of the background spectra recorded just before the introduction of probe molecules.

CO_2_ adsorption capacity was also determined by TG. About 10 mg of samples was pretreated at 120 °C for 1 h under nitrogen atmosphere at 100 ml/min. After cooling down to the adsorption temperature, pure CO_2_ flow was switched in until to the dynamic adsorption equilibrium. The final gained weight was regarded as its CO_2_ adsorption capacity. Saturated samples were regenerated under the pretreatment conditions. This adsorption-regeneration procedure was repeated five times to evaluate the performance stability of modified adsorbent materials.

## Results and Discussion

**Fig. 1:**
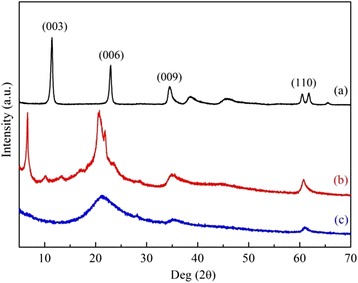
X-ray diffraction profiles of (**a**) LDHs, (**b**) DS/LDHs, and (c) APS/LDHs

**Fig. 2:**
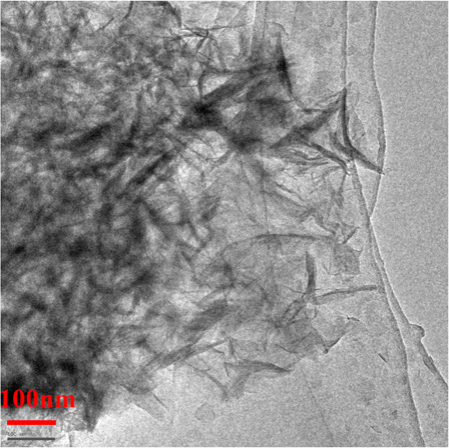
TEM image of APS/LDHs

As it is shown in Fig. 1, there emerged a series of typical peaks due to the layered structures for the as-synthesized LDHs at 2*θ* = 11.5°, 23.0°, 34.5°, and 60.5°, which were ascribed to (003), (006), (009), and (110) lattice planes, respectively, according to the previous literatures [23, 24]. It should be noted that a lower angle Bragg reflection (003), in addition to the well order (00*l*) series, appeared for DS/LDHs generated by the expanded structure as the intercalation of organic anions into the laminated plates of LDHs. This expanded interlayer, suggesting high exposure of surface hydroxyl groups and low diffusion limitation, enabled DS/LDHs to be a precursor for the subsequent introduction of APS. APS grafting reduced the crystallinity dramatically. Yet the hydrotalcite-like structure of the host was conserved for the observed (110) reflection. This was confirmed by the TEM image of APS/LDHs (Fig. 2) as the aggregates of irregular flaky particles were demonstrated.

The molar ratio of C/N is 9 in APS molecule (C_9_H_23_NO_3_Si). Correspondingly, the molar ratios of C/N would be 7, 5, or 3 if 1, 2, or 3 ethoxysilane groups condense with the surface hydroxyl groups on the LDH laminates respectively [21]. Here, the molar ratio of C/N (Table 1) was adopted to further confirm the successful grafting of APS. As the C/N molar ratio of 6.59 was obtained, thus, at least one ethoxysilane group in each APS molecule was tethered to the metal layers while the others presented as the intact ethoxysilane groups or free silanol bonds after hydrolysis.

**Table 1:** Element contents and CO_2_ adsorption properties on APS/LDHs

Sample	N (mmol/g)	C (mmol/g)	Molar ratio of C/N	CO_2_ adsorption capacity (mmol/g)				Amine efficiency			
				25 °C	30 °C	50 °C	75 °C	25 °C	30 °C	50 °C	75 °C
APS/LDHs	3.89	25.62	6.58	1.55	2.05	2.09	1.86	0.40	0.53	0.54	0.48

**Fig. 3:**
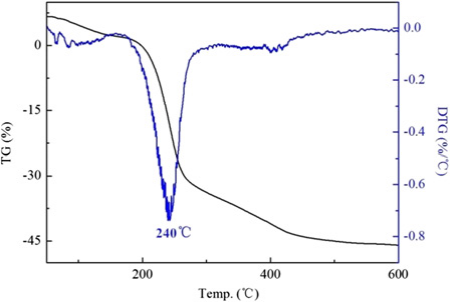
TG-DTG curves of DS/LDHs

**Fig. 4:**
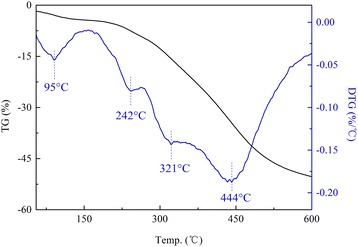
TG-DTG curves of APS/LDHs

**Fig. 5:**
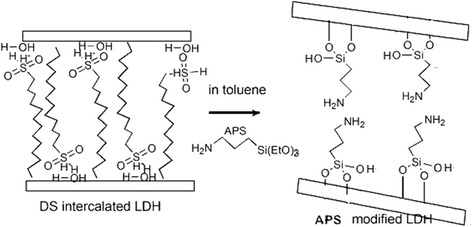
Schematic illustration for the formation of APS/LDHs

TG-DTG (DTG is the derivative curve of TG) study was carried out to investigate the thermal stability of modified LDHs. As it is presented in Fig. 3, DS/LDHs underwent three steps of mass loss, which were ascribed to the removal of adsorbed water below 150 °C; the dehydroxylation during the temperature range from 150 to 300 °C with a prominent loss occurred at 240 °C, as well as the further dehydroxylation and decomposition of dodecyl sulfate over 300 °C [24, 25]. Thermal decomposition behavior of APS/LDHs was significantly different (Fig. 4). It was noted that the weight loss of APS/LDHs due to the dehydroxylation (150~300 °C) was about 10%, much less than that of DS/LDHs (approximately 30%). This mainly could be attributed to the consumption of −OH on the LDH laminates after APS grafting through the condensation reaction as interpreted in Fig. 5. Furthermore, a weight loss peaked at even a higher temperature at 321 °C emerged due to the delayed dehydroxylation on APS/LDHs which might benefit from the hydrogen bonding between the terminal –NH_2_ of APS molecules and the surface hydroxyls on LDH laminates.

**Fig. 6:**
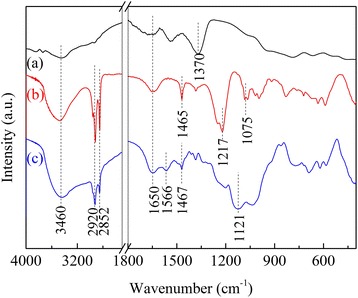
FTIR spectra of (**a**) LDHs, (**b**) DS/LDHs, and (**c**) APS/LDHs

IR spectra of modified LDHs are shown in Fig. 6. For LDHs, the strong broad peak centered at 3460 and the band at 1650 cm^−1^ was related to stretching and bending vibrations of hydroxyl groups from the surface and/or interlayer respectively. The absorption bands at 1370 cm^−1^ were assigned to both monodentate carbonate and intercalated NO_3_^−^ in the interlayer space of LDHs. An overtone of lattice Mg–O vibrations as well as Al–O modes overlay the spectrum ranging from 800 to 400 cm^−1^. The DS molecules in DS/LDHs showed a group of characteristic bands at 2920 (stretching vibration of –CH_3_ groups), 2852 (stretching vibration of –CH_2_ groups), 1465 (C–H bending bond of the organic skeleton), and 1217/1075 cm^−1^ (the asymmetric and symmetric stretching vibrations of –SO_3_^2−^ groups). However, the intensities of these characteristic bands were substantially weakened after the grafting of APS while new bands related to N–H and N–C bonds in APS molecules were observed, e.g., the vibration of N–H_2_ in the primary amine groups (RNH_2_) at 1570/1468 cm^−1^ and the C–N bending vibration of C–N at 1124 cm^−1^ [13, 23].

Even though part of surface −OH groups on LDH laminates were consumed through its condensation reaction with the silanols of APS as it was abovementioned, the bands located at 3460 and 1650 cm^−1^ were somewhat enhanced. It was reported that non-uniform distribution of grafted APS would occur, like clustering of amino groups via their intermolecular hydrogen bonding [16] and protonation of amine species (RNH_3_^+^) generated under the existence of water [26], with surface hydroxyls or with free silanol groups. It was also held that the basic molecules or groups could be hydrogen-bonded onto the surface hydroxyls of LDHs even though this bonding was rather weak [27]. Thus, here, the enhancement of these absorption bands was attributed to the bounded –NH_2_ groups overlapped with the –OH group at the same IR region [28], indicating a rather different local environment for CO_2_ adsorption.

**Fig. 7:**
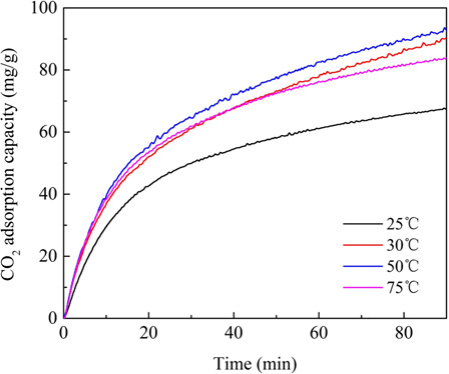
CO_2_ adsorption profiles on APS/LDHs at different temperatures

CO_2_ adsorption performance of amine-modified LDHs was investigated by TGA at different testing temperatures. As it is shown in Fig. 7, CO_2_ adsorption amounts on APS/LDHs increased dramatically within the first 30 min, followed by a slow saturation stage. A CO_2_ adsorption capacity as high as 2.09 mmol/g during this saturation stage was obtained, much higher than that of LDHs (typically less than 1.0 mmol/g [29–31]). Therefore, the incorporated amino groups contributed to CO_2_ capturing significantly. It should be noted that APS/LDHs exhibited a CO_2_ adsorption amount of 1.55 mg/g at 25 °C while it showed a rather stable adsorption capacity at a temperature range from 30 to 75 °C. APS begins to react with CO_2_ from 28 °C [21]. Thus, the higher viscosity of APS at 25 °C would generate greater mass transfer limitation and further weaken its function for CO_2_ capturing.

It was the accessibility of amino groups and the proximity of adjacent amino pairs that essentially determined the CO_2_ adsorption capacity for amine-modified materials. The impregnated amines would like to agglomerate in the pores of supports which generated strong diffusion limitation for CO_2_ molecules from surface into bulk [2, 9, 25, 32]. However, the accessibility of active sites in APS/LDHs might also be affected adversely by the hydrocarbon chains intercalated between the LDH laminates, which reduced the mobility and relative proximity of amino pairs. This was unfavorable to the amine efficiency of APS/LDHs presumably, which yet turned out to be slightly higher than 0.5 that was the maximum value based on the zwitterion mechanism (Table 1). Thus, it was deduced there may be a different adsorption mechanism here contributing to the amine efficiency.

**Fig. 8:**
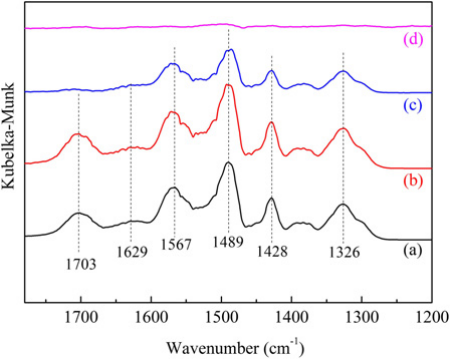
DRIFTS spectra of CO_2_ adsorption and desorption on APS/LDHs: (**a**) adsorption for 5 min, (**b**) adsorption for 20 min, (*c*) purge for 30 min at room temperature, (**d**) purge for another 30 min at 120 °C

DRIFTS measurement was adopted to investigate the CO_2_ adsorption mechanism on APS/LDHs (Fig. 8). Exposure to CO_2_/N_2_ gas mixture led to the appearance of several typical absorption peaks, which were due to the N–H deformation in RNH_3_^+^ at 1629 and 1489 cm^−1^, the asymmetric stretching mode of COO^−^ at 1567 cm^−1^, and the NCOO skeletal vibration at 1428 and 1326 cm^−1^ [13]. Obviously, CO_2_ adsorption onto the primary amines happened via the zwitterion mechanism with a two-step sequence, i.e., the formation of a zwitterion firstly and the subsequent proton transfer [13, 14].

Especially, the emerged band at 1703 cm^−1^ needed further identification. As it disappeared completely under mild regeneration conditions while the generated carbamates decomposed under a higher temperature, this band might be related to a weakly bound CO_2_ species around the bounded −NH_2_ group [33] which provided a different local environment for adsorption. CO_2_ molecules could be captured onto these amino groups via hydrogen bonding. Wu et al. [13] attributed the absorption band at 1706 cm^−1^ to the hydrogen-bonded CO_2_ species generated by its adsorption near the protonated amine groups. Danon et al. [16] also found that the SBA-15 surface play a significant role in the specific interactions between CO_2_ and moieties tethered to the surface of SBA-15.

**Fig. 9:**
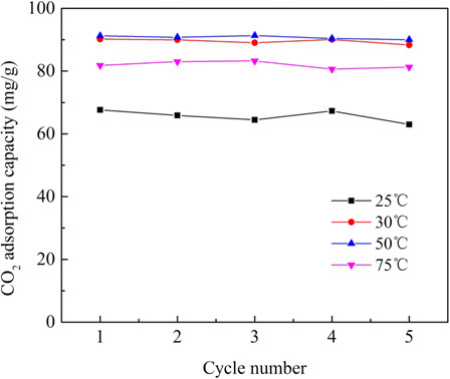
Performance stability of APS/LDHs during the consecutive cycles of adsorption-desorption

Stable cyclic adsorption/desorption performance of adsorbents is especially desired for practical separation process. The cyclic performance of APS/LDHs was obtained (Fig. 9) through its exposure to CO_2_ at different adsorption temperatures and then regeneration at 120 °C repeatedly. The CO_2_ adsorption capacity fluctuated around its initial adsorption amount during these five cycles at these testing temperatures, showing excellent cyclic performance. This provided APS/LDHs great feasibility to capture CO_2_ from fossil fuel-based thermal power plants [1]. Firstly, energy saving for regeneration could be achieved compared with the calcined LDH samples that needed activation at elevated temperatures, e.g., 400 °C [34]. What is more, the CO_2_ adsorption on calcined LDH samples decreased with the increasing number of thermal adsorption/desorption cycles due to irreversible chemisorption caused by the poor thermal stability and agglomeration of particles [35], while APS/LDHs here showed superior performance stability.

## Conclusions

A monolayer of terminal amino groups of APS was grafted onto LDHs (APS/LDHs) for CO_2_ capture. It was found that the enlarged layer spacing pillared by dodecyl sulfonate was beneficial to APS introduction. APS was tethered to the LDH laminates through covalent interaction. The incorporated amino groups contributed a lot to CO_2_ capturing on APS/LDHs both through the zwitterion mechanism and weak bonding as confirmed by DRIFTS results. The CO_2_ adsorption capacity stabilized at about 90 mg/g during the five cycles of adsorption-desorption, showing a great application potential in the temperature swing adsorption processes.

For LDH-based oxide adsorbents, the gradually reduced CO_2_ uptakes could be observed primarily due to the increasingly lack of availability of the basic sites. However, APS/LDHs are more robust to a variety of treatment conditions as it based on the chemical combination of (3-aminopropyl)triethoxysilane and the metal layers. This effectively prevents the significant loss of adsorption capacity due to the organic leaching out of the solids during the cyclic testing. And the captured CO_2_ could be completely desorbed under 120 °C on APS/LDHs, which is a rather safe operation temperature to avoid the amine degradation or thermal conformational alteration.

## Abbreviations

APS: (3-Aminopropyl)triethoxysilane
DRIFTS: Diffuse reflectance infrared Fourier transform spectroscopy
DS: Dodecyl sulfate
EA: Elemental analysis
FTIR: Reflectance Fourier transform infrared spectrometer
HMS: Hexagonal mesoporous silica
LDHs: Layered double hydroxides
TEM: Transmission electron microscopy
TG-DTG: Thermogravimetric analysis-derivative curve of TG
XRD: X-ray diffraction
